# Extensive rib resection followed by thoracic wall reconstruction using polytetrafluoroethylene mesh and titanium plates for refractory intercostal artery bleeding induced by severe blunt thoracic injury: report of a case

**DOI:** 10.20407/fmj.2020-026

**Published:** 2021-03-20

**Authors:** Yosuke Kobayashi, Shokei Matsumoto, Kosuke Tajima

**Affiliations:** 1 Department of Surgery, Tokyo Saiseikai Central Hospital, Minato, Tokyo, Japan; 2 Department of Trauma and Emergency Surgery, Saiseikai Yokohama-shi Tobu Hospital, Yokohama, Kanagawa, Japan; 3 Emergency Department, Fujita Health University Hospital, Toyoake, Aichi, Japan

**Keywords:** Massive hemothorax, Blunt chest trauma, Multiple rib fractures, Intercostal artery injuries, Thoracic wall reconstruction

## Abstract

Massive hemothorax due to multiple rib fractures and intercostal artery (ICA) injuries is one of the most lethal forms of chest trauma. Urgent thoracotomy is required; however, suturing is sometimes difficult owing to the limited operative field in the thoracic cavity and because the transected ICA retracts between the surrounding intercostal muscles. We present a patient with refractory ICA bleeding induced by severe blunt thoracic injury successfully treated with extensive rib resection followed by thoracic wall reconstruction using GORE^®^ DUALMESH^®^ and titanium plates. A 66-year-old woman attempted suicide by diving into the path of a train. She incurred massive left hemothorax associated with multiple rib fractures and severe trauma to her extremities; both upper limbs and left leg at the thigh were nearly disconnected. Initially, she underwent urgent left anterolateral thoracotomy followed by partial lung resection and suture hemostasis of the thoracic wall. Subsequently, interventional radiology was performed for the ICA bleeding, and her extremities except her right leg were amputated. However, because hemothorax persisted, and because of the comminuted fractures, we removed the fifth to eighth ribs, and the ICA vascular sheath was ligated. Resecting multiple ribs caused deformities and lung herniations, although hemostasis was achieved. On the third postoperative day, thoracic reconstruction using Gore-Tex^®^ Dual Mesh and titanium plates was performed. Although a small empyema occurred, it was controlled with antibiotics and drainage. Paradoxical respiration and atelectasis did not occur, and the patient was moved to the hospital for continued care in a lucid state.

## Introduction

Rib fractures with intercostal artery laceration and lung laceration due to blunt thoracic trauma can cause massive hemothorax on rare occasions.^1^ Resecting crushed rib fragments may be effective for hemostasis in life-threatening chest wall bleeding if sutures fail; however, a large defect may also occur. We herein report a case of refractory intercostal artery (ICA) bleeding induced by severe blunt thoracic injury successfully treated with extensive rib resection followed by thoracic wall reconstruction using Gore-Tex^®^ Dual Mesh and titanium plates.

## Case Presentation

A woman in her 60s who attempted suicide by diving into the path of a train arrived at our hospital in a hemodynamically unstable condition. Left massive hemothorax was detected, and initial bleeding was 1000 ml after tube drainage. She was transferred to the operating room with life-threatening injuries. Both upper limbs and her left leg at the thigh were nearly disconnected, and she also suffered a right femoral neck fracture. Left anterolateral thoracotomy was performed, and bleeding was detected from the lung and the thoracic wall. Partial resection and ligation of the lung was performed for hemostasis; however, it was impossible to completely stop the bleeding from the multiple posterior rib fractures. Following amputation of both upper limbs and the left leg, secondary abdominal compartment syndrome occurred owing to a necessary massive transfusion. Open abdominal management was initiated with vacuum pack closure. In addition, venovenous extracorporeal membrane oxygenation was introduced to improve severe hypoxemia. Interventional radiology (IVR) was attempted; however, the ICAs were spastic, and the procedure failed. The patient was transferred to the intensive care unit (ICU) with the expectation that her bleeding would be self-limited. However, hemothorax worsened several hours later, and her condition became life-threatening once again. Emergency thoracotomy was performed in the ICU, and the incision was extended laterally and posteriorly in the right lateral decubitus position owing to cardiopulmonary arrest. ICA bleeding was identified between the fifth and the eighth ribs, but the bleeding could not be controlled by electrocautery, compression, and suturing. Crushed ribs from the fifth to the eighth intercostal space were removed, and the ICA vascular sheath was ligated at the same level in approximately 5 min. Hemostasis was obtained, and damage control was achieved by packing the thoracic cavity with gauze and placing a running suture around the wound margin, because coagulopathy was present.

**Figure 1:**
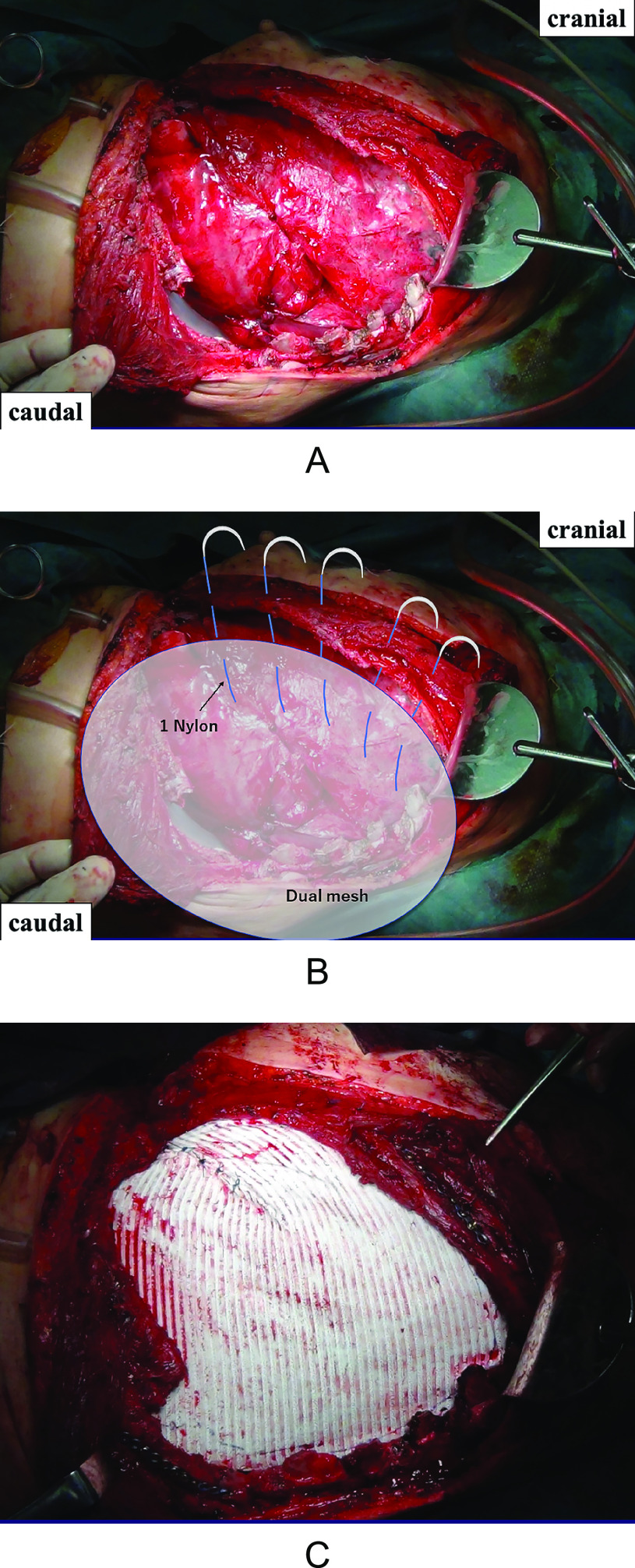
A) The crushed fifth to eighth ribs were removed, and the intercostal artery vascular sheath was ligated at the same level to achieve hemostasis. The large anterolateral chest wall defect measured 30 cm×15 cm. B) Gore-Tex^®^ Dual Mesh was sutured with an approximately 1-cm margin from the edge, pulling the mesh with adequate tension, and trimming step-by-step with fixation. C) The thoracic wall was reconstructed using Gore-Tex^®^ Dual Mesh and titanium plates. The inlay of the Dual Mesh was sutured to the thorax, and titanium plates were used for fixation of the deviated fourth and ninth ribs.

On the third postoperative day, the gauze in the thoracic and abdominal cavity was removed, and homeostasis was confirmed. The thoracic wall defect from the fifth to the eighth rib measured 30 cm×15 cm (Figure 1A). This area was covered with 2-mm Gore-Tex^®^ Dual Mesh, and size 1 nylon was used to suture the remaining thoracic wall edges (Figure 1B). The deviated and unstable fourth and ninth ribs were fixed with 2-mm titanium mandibular locking plates (Synthes MLP; Synthes Maxillofacial, Paoli, PA) (Figure 1C). A 32-French chest tube and a 19-mm J-VAC drain were inserted, and the chest was closed.

**Figure 2:**
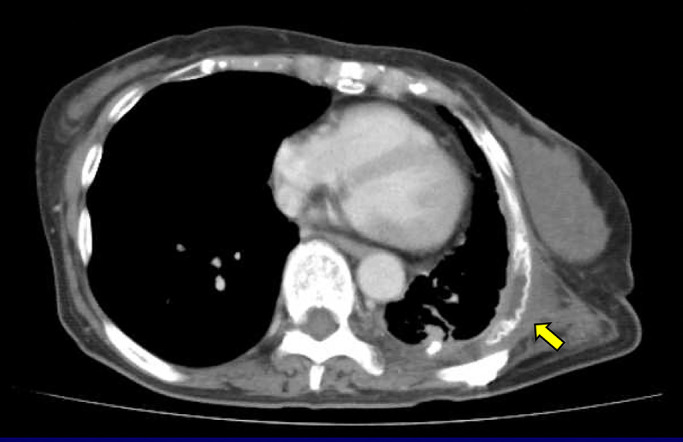
Computed tomography 3 months after the reconstruction showing good lung expansion with no atelectasis despite seroma formation. The yellow arrow indicates the Dual Mesh.

Although a small empyema occurred, it resolved with antibiotics and drainage. Paradoxical respiration and atelectasis did not occur (Figure 2), and the patient was moved to the hospital for continued care in a lucid state 85 days after surgery.

## Discussion

Chest wall bleeding rarely requires intervention; however, bleeding may be difficult to control surgically, specifically when it occurs because of posterior rib fractures. The basic surgical hemostatic technique is figure-of-eight suture ligation of the bleeding ICA at the inferior border of the rib, where the injury occurred. An alternative method involves suturing to encircle a rib lateral to the bleeding point and compressing the vessel against the inferior groove with the encircling suture.^2^ However, these techniques are extremely difficult in cases with multiple posterior rib fractures. Recently, the feasibility and reliability of transcatheter arterial embolization have been demonstrated to be beneficial in controlling bleeding from an injured ICA.^3–5^ Endovascular aortic stenting has also been used successfully to control ICA bleeding.^6^ In our case, we had planned to manage the chest wall bleeding with IVR when the bleeding could not be controlled via the usual surgical methods; however, the ICAs were spastic, and IVR failed. Subsequently, chest bleeding was so intense and life-threatening that a bedside emergency resuscitative thoracotomy was necessary. Bleeding was not controlled by electrocautery, compression, and suturing. Diffuse bleeding was identified from the chest wall surrounding the lateral side of the fifth to the eighth ribs, but the exact location could not be confirmed. Multiple posterolateral ribs had been destroyed, and bleeding points were unclear; therefore, it was necessary to remove these ribs to stop the bleeding. After resection, the ICA sheath was ligated, and hemostasis was achieved.

Cutaneous muscle flaps have been used traditionally to reconstruct large chest wall defects after chest trauma.^7^ However, such lengthy and cumbersome surgeries should never be considered in the initial care of trauma patients with life-threatening conditions. It is recommended that quicker and easier techniques be employed in such cases. It is possible to reconstruct various sections of the chest wall using Gore-Tex^®^ Dual Mesh, and this technique is now widely accepted for chest wall defects after surgery in patients with chest wall tumors.^8–10^ This product is a pure and unique polytetrafluoroethylene (ePTFE) consisting of two functionally distinct surfaces. The smooth surface is designed for minimal tissue attachment, whereas the patterned, indented surface is designed for active tissue attachment. The mesh is easily manipulated and stretched, and is rigid enough to prevent paradoxical chest wall motion. The mesh can also be used to create a watertight closure. We chose Gore-Tex^®^ Dual Mesh because of these features; however, there are few reports of thoracic wall reconstruction using synthetic mesh following chest trauma.

Synthetic mesh has a history of safe and effective use in hernia surgeries. However, among several potential complications with mesh, infection is the most serious. Although mesh infection is rare with elective surgeries, there is a fear that acute-phase trauma patients have a greater chance of developing infection.^11^ If a synthetic mesh infection occurs, mesh removal is recommended although there are a few reports of conservative therapy for mesh infection.^12,13^ In our case, chest wall reconstruction was performed immediately after the patient’s hemodynamics stabilized, to achieve early extubation and to prevent postoperative infection. Minor mesh infection occurred in the wound and in the chest cavity; however, these were successfully treated conservatively with drainage and antibiotics.

In conclusion, we demonstrated the feasibility of thoracic wall reconstruction using Gore-Tex^®^ Dual Mesh and titanium plates. If a trauma patient has a chest wall defect, this technique may be considered because it is easy to perform, the materials are readily available, and the approach may be effective in such cases. However, further research analyzing outcomes such as the need for chest wall reconstruction and the incidence of paradoxical respiration and chest wall hernia after chest wall defect repair should be conducted before widespread adoption of this technique.
